# Structural Insights into the Interactions of Digoxin and Na^+^/K^+^-ATPase and Other Targets for the Inhibition of Cancer Cell Proliferation

**DOI:** 10.3390/molecules26123672

**Published:** 2021-06-16

**Authors:** Yulin Ren, Sijin Wu, Joanna E. Burdette, Xiaolin Cheng, A. Douglas Kinghorn

**Affiliations:** 1 Division of Medicinal Chemistry and Pharmacognosy, College of Pharmacy, The Ohio State University, Columbus, OH 43210, USA; wusj@dicp.ac.cn (S.W.); cheng.1302@osu.edu (X.C.); 2 Department of Pharmaceutical Sciences, College of Pharmacy, University of Illinois at Chicago, Chicago, IL 60612, USA; joannab@uic.edu

**Keywords:** digoxin, cytotoxicity, Na^+^/K^+^-ATPase, docking profiles, molecular targets

## Abstract

Digoxin is a cardiac glycoside long used to treat congestive heart failure and found recently to show antitumor potential. The hydroxy groups connected at the C-12, C-14, and C-3′a positions; the C-17 unsaturated lactone unit; the conformation of the steroid core; and the C-3 saccharide moiety have been demonstrated as being important for digoxin’s cytotoxicity and interactions with Na^+^/K^+^-ATPase. The docking profiles for digoxin and several derivatives and Na^+^/K^+^-ATPase were investigated; an additional small Asn130 side pocket was revealed, which could be useful in the design of novel digoxin-like antitumor agents. In addition, the docking scores for digoxin and its derivatives were found to correlate with their cytotoxicity, indicating a potential use of these values in the prediction of the cancer cell cytotoxicity of other cardiac glycosides. Moreover, in these docking studies, digoxin was found to bind to FIH-1 and NF-κB but not HDAC, IAP, and PI3K, suggesting that this cardiac glycoside directly targets FIH-1, Na^+^/K^+^-ATPase, and NF-κB to mediate its antitumor potential. Differentially, digoxigenin, the aglycon of digoxin, binds to HDAC and PI3K, but not FIH-1, IAP, Na^+^/K^+^-ATPase, and NF-κB, indicating that this compound may target tumor autophagy and metabolism to mediate its antitumor propensity.

## 1. Introduction

Cancer has the properties of aggressive cellular growth, relapse, metastasis, and resistance to the existing cancer chemotherapeutic agents. They are often genetic diseases, and there is a mutator phenotype found ubiquitously in cancer cells. Thus, in theory, those agents designed based on molecular targets but not cancer cells themselves could be effective in treating cancers in general [1,2].

Na^+^/K^+^-ATPase (NKA) is a P-type ion-translocating ATPase activated by Na^+^ and K^+^ and found in all mammalian cells. This enzyme was proposed in 1957 by the Danish scientist Prof. Jens C. Skou, who shared the Nobel Prize in Chemistry in 1997, “For the first discovery of an ion-transporting enzyme, Na^+^/K^+^-ATPase” [3,4]. The two-dimensional crystals in pure membrane-bound NKA were reported in 1981 [5]; the first view of the entire heterodimer of α and β subunits was demonstrated in 2001, based on a structure of the E2-state NKA at 11-Å resolution [6]. The crystal structures of NKA showed that this protein contains the catalytic α-subunit (α1, α2, α3, and α4 isoforms in human), the heavily glycosylated β-subunit (β1, β2, and β3 isoforms in human), and the tissue-specific auxiliary regulatory subunit, FXYD (the isoform expressed in kidney called as the γ-subunit). Of these, the α-subunit consists of three cytoplasmic domains, A (actuator), N (nucleotide binding), and P (phosphorylation), and ten transmembrane helices (M1–M10). In addition, NKA has E1 and E2 conformational states, in which the transmembrane cation-binding sites in the E1 state have a high affinity for Na^+^, and those in the E2 state have a high affinity for K^+^ [7,8]. The crystal structures of the complexes of NKA, and ouabain and digoxin, showed that these cardiac glycosides bind mainly to the transmembrane helices αM1–αM6 of the catalytic α-subunit of NKA [9,10].

NKA pumps three Na^+^ ions out and two K^+^ ions into the cell in each pump cycle, and thus creates and maintains gradients of Na^+^ and K^+^ ions across the plasma membrane, which are required for various cellular activities, including cell growth, differentiation, and survival. Thus, blocking NKA causes intracellular K^+^ depletion and a Ca^2+^ increase, which induces apoptosis and triggers excitotoxicity of necrotic cell death, respectively [11], and dysfunction of this pump is responsible for some pathologies [12]. Recently, NKA has been determined as an anticancer drug target, as evidenced by the increasing number of antitumor NKA inhibitors, including cardiac glycosides [13,14]. However, the effects of cardiac glycosides on NKA are not yet fully elucidated. For example, several cardiac glycosides activate NKA at low concentrations but inhibit this protein at high concentrations [15]. Thus, examination of the interactions of cardiac glycosides and NKA could be supportive of a better understanding of the mechanisms of the antitumor activity of these compounds.

It has been well documented that the cardiotonic steroid-binding sites of NKA play important roles in regulating physiological processes [16], and the saccharide moiety and steroidal hydroxy groups were found to be required for cardiac glycosides to bind to NKA [17]. In addition, the antimigratory effects were found to correlate directly with the inhibition of NKA when ouabain and its derivatives were tested against human breast cancer cells [18], and similar evidence was observed in recent investigations on digoxin and (+)-strebloside [19,20]. These prior studies indicate some promise for the design of antitumor cardenolides by targeting NKA.

Digoxin, a well-known cardiac glycoside used medicinally to treat congestive heart failure, was found recently to show potential antitumor activity by inhibiting NKA, hypoxia-inducing factor 1α (HIF-1α), and nuclear factor kappa B (NF-κB) activation, and by induction of autophagy and immunogenic cell death [19,21]. However, digoxin is toxic and has a narrow therapeutic index that may result from its NKA inhibition, and this hinders its use in cancer chemotherapy [22]. Thus, the current interest in digoxin has switched to its synthetic modifications and its interactions with NKA, as exemplified by a new derivative of digoxin, which has a γ-benzylidene group substituted at the C-17-butenolide, which exhibited more potent cytotoxicity and lower NKA inhibitory activity than digoxin itself [23]. Interestingly, several analogues of digoxin were found to interact with the NKA isoforms specifically and to modulate NKA selectively [24], indicating that these synthetic derivatives could target NKA differentially to mediate their cytotoxicity. These different interactions may overcome the narrow therapeutic index observed in the clinical use of digoxin.

However, to the best of our knowledge, detailed information about the interactions of synthetic derivatives of digoxin and NKA has not been reported thus far, and the binding between digoxin and other molecular targets is not well demonstrated in the literature. Thus, presented herein are the detailed interactions of NKA and digoxin or its derivatives (**3**–**11**), in which digoxin has been changed at its steroid hydroxy groups, the lactone unit, and the saccharide moiety. We also discuss a correlative link between the docking scores from the binding to NKA and the cytotoxicity of these cardenolides; and the docking profiles for digoxin or digoxigenin and histone deacetylase (HDAC), factor inhibiting HIF-1 (FIH-1), inhibitor of apoptosis (IAP), NF-κB, and phosphoinositide 3-kinase (PI3K).

## 2. Results

### 2.1. Structural Model of NKA and Modeling Validation

The cytotoxicity against human cancer cells of digoxin and selected synthetic derivatives, and the inhibitory effect of digoxin on swine NKA, have been reported in a previous investigation [19]. In the present molecular modeling study, the crystal structure of *Sus scrofa* NKA (sNKA, with 1021 amino acids) obtained from the Protein Data Bank (PDB) database (https://www.rcsb.org/pdb, accessed on 2 March 2021) [25] was selected as the template. A human NKA (hNKA with 1023 amino acids) homology model has been built [19,20], which has been used to predict the binding conformations and affinities of digoxin derivatives and NKA.

In structure modeling, alignment of the query protein’s sequence to that of the template is critically important, and thus a sequence consistency scheme for the hNKA model and the sNKA template was generated, using the Clustal X version 2.0 [26]. As shown in Figure S1, a high degree of similarity was observed in the local alignment of essential functional domains of hNKA and sNKA. A total of 1013 amino acids of hNKA and sNKA were found to be similar, with the positive ratio reaching 99% (1013/1023); 1004 amino acids of these two proteins are identical, with the identity being up to 98% (1004/1023). Two redundant residues of hNKA, Gly23 and Lys24, are located at the N-terminal, which have not been observed in any crystal structures of NKA reported thus far. In addition, the transmembrane domain of NKA focused on herein is more conserved than the intracellular nucleotide-binding domain, and the most varied residues were found far away from the ion-binding site and transport pathway comprised of αM1–αM6 helices. Therefore, it is suitable for building a hNKA model based on a sNKA template, for inspection of the binding between digoxin or its derivatives and NKA.

All of the nine sNKA structures found in the PDB database are in the either E1P or E2P state, but both the conformations and binding affinities for Na^+^ and K^+^ ions were found to be different in these two states, as shown in the complex crystal structures reported for Na^+^- and bufalin-bound NKA in E1P and E2P states, respectively (Figure S2) [10,27]. In the E2P state, NKA has a high affinity for K^+^ and imports it into the cytoplasm, with the cavity of the upper extracellular part of the transport pathway being much greater than that in the E1(P) state. This large space could support a cardiac glycoside to bind to and inhibit NKA, and thus four sNKA structures in the E2P state that are co-crystallized with cardiotonic steroids, including 3N23 (ouabain), 4HYT (ouabain), 4RES (bufalin), and 4RET (digoxin), were selected for the hNKA modeling in this investigation.

**Figure 1:**
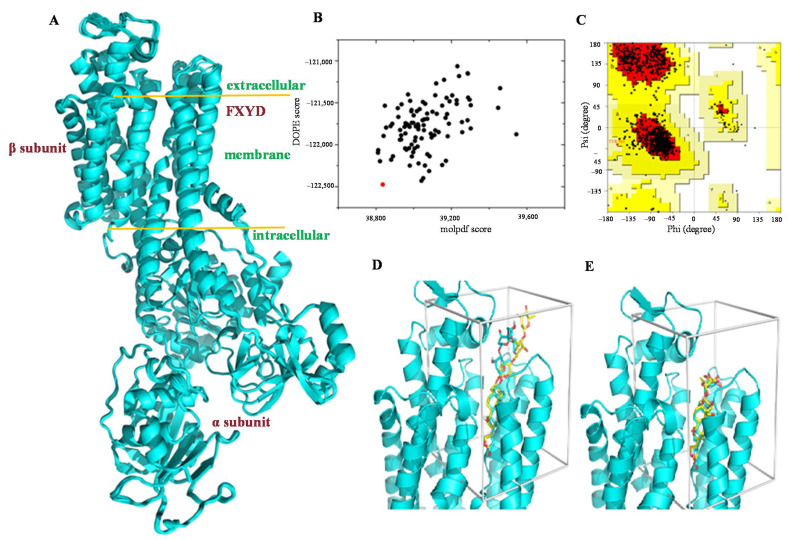
Modeling of hNKA. (**A**) Structural alignment of 30 modeling results for hNKA. The structures were built by Modeller 9.10 and evaluated and filtered by DOPE, molpdf scores, and Ramachandran plot. (**B**) Plot of DOPE and molpdf scores. The red dot represents the selected model. (**C**) The Ramachandran plot of the selected structure. The red, yellow, and light-yellow regions represent the most favored, allowed, and generally allowed regions, respectively, as defined by ProCheck. (**D**,**E**) Docking results of digoxin (**D**) and ouabain (**E**). The binding conformations of digoxin and ouabain in the corresponding co-crystal structures were reproduced for the hNKA model by Autodock VINA. Shown in cyan are those built with the hNKA model; shown in yellow are those from the crystal structures of PDB 4RET (digoxin-bound, **D**) and PDB 4HYT (ouabain-bound, **E**).

Altogether, 100 structures were built by Modeller 9.10, and three different criteria were used to evaluate and to filter the modeling results (Figure 1A), including discrete optimized protein energy (DOPE) [28], molpdf scores, and Ramachandran plots (Figure 1B,C) [29,30]. The model that displayed the lowest DOPE and molpdf scores was selected. Based on the Ramachandran plot analysis, up to 87.8% of residues of this model appeared in the most favored regions, with around 12.1% residues being situated in the allowed regions. Only 0.1% of the residues were observed in the generally allowed regions, with no residues being located in the disallowed regions (Table S1). This selected model was validated further by comparison of the docking profiles for digoxin or ouabain with those observed from the co-crystal structures of NKA. The docking conformations of digoxin and ouabain with the hATPase model, root-mean-square deviation (RMSD) 0.134 Å for digoxin and 0.422 Å for ouabain between the steroid and lactone groups, are quite consistent with those observed from the co-crystal structures of these compounds with NKA (Figure 1D,E), indicating that this hNKA model could be used as a receptor structure for the docking investigation on digoxin and its derivatives.

### 2.2. Impact of the Hydroxy Groups on the Binding between Digoxin and hNKA

As a well-known molecular target, NKA was found to interact with the U-shaped steroid core, the β-oriented C-14 hydroxy group, the C-17 unsaturated lactone unit, and the C-3 carbohydrate moiety of each cardiac glycoside [13,17], including digitoxin (**1**) and digoxin (**2**) [19,31]. The crystal structure of the pig kidney NKA (α1β1γ) and digoxin (**2**) showed that hydrogen bonds may be formed between the HO-12β and HO-14β of **2** and the Asn122 (αM2) and Thr797 (αM6) side chains of NKA, respectively, with its first glycosyl group and the 17β-lactone unit being located in a wide cavity and a hydrophobic funnel of the target, respectively [10]. Thus, the HO-12β, HO-14β, and 3β-glycosyl groups and the 17β-attached lactone unit of **2** all seem to be important for its binding to NKA.

**Figure 2:**
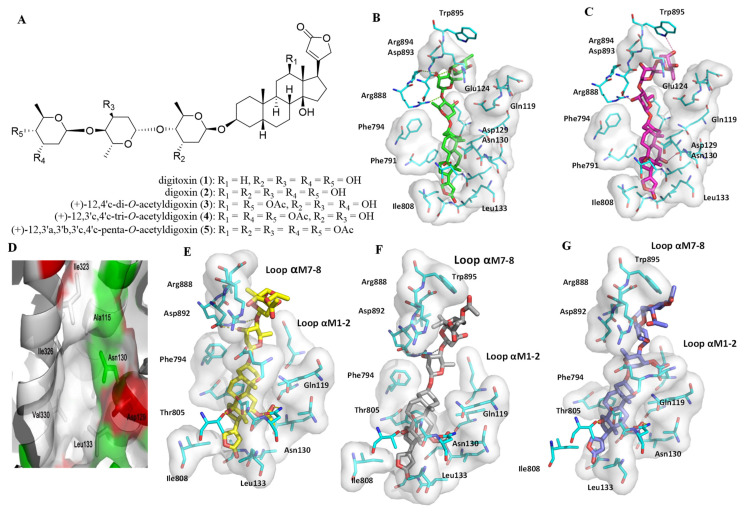
Docking profiles for digitoxin (**1**), digoxin (**2**), (+)-12,4′c-di-*O*-acetyldigoxin (**3**), (+)-12,3′c,4′c-tri-*O*-acetyldigoxin (**4**), and (+)-12,3′a,3′b,3′c,4′c-penta-*O*-acetyldigoxin (**5**) with NKA. (**A**) Structures of **1**–**5**. (**B**,**C**) Docking profiles for **1** (green, **B**) and **2** (pink, **C**) with NKA. (**D**) The Asn130 pocket that C-12 position targeted (white, residues with hydrophobic sidechains; green, residues with polar sidechains; red, residues with negatively charged sidechains). (**E**–**G**) Docking profiles for **3** (yellow, **E**), **4** (gray, **F**), or **5** (blue, **G**) and NKA.

To test the role of hydroxy groups of **2** in its interaction with hNKA, the computer simulated docking profiles for digitoxin (**1**), digoxin (**2**), or the acetylated derivatives of **2**, including (+)-12,4′c-di-*O*-acetyldigoxin (**3**), (+)-12,3′c,4′c-tri-*O*-acetyldigoxin (**4**), and (+)-12,3′a,3′b,3′c,4′c-penta-*O*-acetyldigoxin (**5**) (Figure 2A), and hNKA were investigated; and the docking scores and cytotoxicity toward human cancer cells are presented in Table 1.

As shown in Figure 2B,C and Figure S3A, compounds **1** and **2** bind similarly to hNKA, but the hydroxy substituent at the C-12 position of **2** points to a small pocket on the side of the binding cavity, namely, the Asn130 pocket. This pocket is located between αM2 and αM4, with αM1 helix being at the bottom. It is composed of several hydrophobic residues, including Ala115, Ile323, Ile326, Leu133, and Val330, with a polar residue, Asn130, being located in the deep part (Figure 2D). Digitoxin (**1**) has little interaction with this side pocket, owing to the absence of any substituents at the C-12 position, but the C-12 hydroxy group of **2** can reach the entry part of the Asn130 pocket. However, a free energy penalty may occur due to the hydrophobic nature of other residues in the pocket, which might negatively affect the binding affinity of **2**. Additionally, this penalty impact seems to be greater than other interactions between HO-12β of **2** and the Asn130 pocket of hNKA. Thus, **2** showed less binding affinity and cancer cell cytotoxicity when compared with **1** (Table 1).

Compounds **3**, **4** and **5** have the same steroid core, but acetoxy groups are substituted differentially at their saccharide units. The second and third saccharide moieties are located outside the cation pocket of hNKA and seem not to affect the binding affinity greatly (Figure 2E–G and Figures S3 and S4). However, the first saccharide unit inserts into the cation pocket, and substituting it could have significant impacts on the interactions of these compounds and hNKA. Consequently, compounds **3** and **4** that have acetoxy groups at the terminal saccharide moiety showed similar binding affinities and cytotoxic potencies to one another, which are greater than those of **5**, which has acetoxy groups substituted at the first, second, and terminal saccharide moieties (Table 1) [19].

In the binding poses of **3**, **4** and **5**, an acetoxy group at the C-12 position would insert deeper into the Asn130 pocket than the C-12 hydroxy group of **2**, to support the formation of a hydrogen bond between this acetoxy group and the amino group of the Asn130 sidechain. Accordingly, this acetoxy group could benefit the binding between these compounds and hNKA. However, as shown in Figure S3, the substitution of acetoxy groups changed the conformation of the glycosyl groups of **3**–**5**, when compared with that of **2**, and this change could in turn decrease the binding between these compounds and hNKA. In addition, the acetoxy groups at the terminal saccharide of **5** tend to interact with the loop αM7–αM8 of hNKA, which could result in the other saccharide moieties, especially the second one, moving away from the loop αM1–αM2, the key part for cardiac glycosides’ binding to NKA [34]. Therefore, the binding affinities and cancer cell cytotoxic potencies of **3**–**5** are weaker than those of **2** (Table 1).

Thus, digoxin binds to hNKA, at least in part, by the hydroxy groups connected at its steroid core and the glycosyl groups. These groups interact directly with hNKA through hydrogen bonds or the interactions with the hydrophobic residues in the cationic pockets of hNKA, and any of their substitutions could potentiate or de-potentiate the binding of digoxin and hNKA.

### 2.3. Impact of the Lactone Unit on the Binding between Digoxin and hNKA

**Figure 3:**
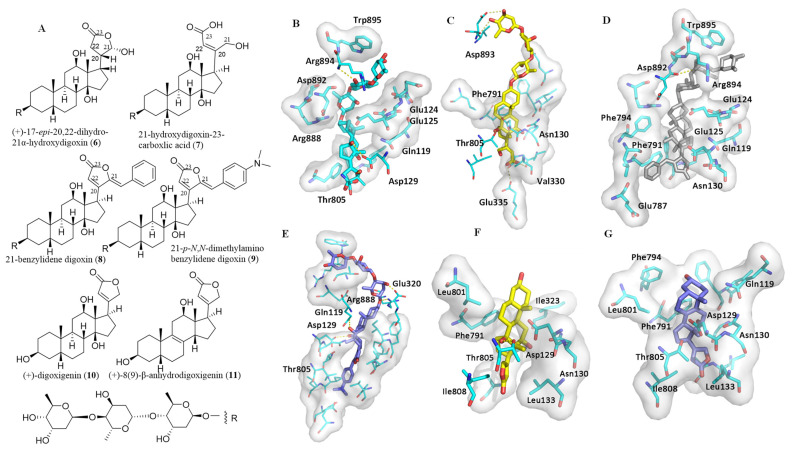
Docking profiles for (+)-17-*epi*-20,22-dihydro-21α-hydroxydigoxin (**6**), 21-hydroxydigoxin-23-carboxlic acid (**7**), 21-benzylidene digoxin (**8**), 21-*p*-*N*,*N*-dimethylaminobenzylidene digoxin (**9**), (+)-digoxigenin (**10**), and (+)-8(9)-β-anhydrodigoxigenin (**11**) with NKA. (**A**) Structures of **6**–**11**. (**B**–**G**) Docking profiles for **6** (cyan, **B**), **7** (yellow, **C**), **8** (gray, **D**), **9** (blue, **E**), **10** (**F**, yellow), and **11** (blue, **G**) with NKA.

To test the importance of the C-17 unsaturated heterocycle unit, the binding between digoxin derivatives containing different substituents at the C-17 position and hNKA was inspected. These included (+)-17-*epi*-20,22-dihydro-21α-hydroxydigoxin (**6**), 21-hydroxydigoxin-23-carboxlic acid (**7**), 21-benzylidene digoxin (**8**), and 21-*p*-*N*,*N*-dimethylaminobenzylidene digoxin (**9**) (Figure 3A–E).

Compared with **2**, **6** has a different configuration at the C-17 position, and thus, as discussed previously [19], it seems not to fit into the cation pocket of hNKA. The lactone unit of **6** rotates away from the cationic binding site of hNKA, which makes the carbonyl group orient toward a small groove on the side of the pocket. However, the HO-21α substituent impairs the binding of **6** to the hydrophobic groove. Additionally, the rotated lactone unit results in a slight rotation of its steroid core, which breaks the hydrogen bonds between **6** and hNKA (Figure 3B). Thus, **6** shows poor binding affinity and cytotoxicity (Table 1).

The lactone unit of **7** is replaced by a hydroxybutenoic acid, which makes **7** bind to hNKA in a pose similar to that of **2** (Figure 3C and Figures S3 and S4). Thus, **7** can insert deeply into the cation-binding pocket to form one or two hydrogen bonds with the Glu335 sidechain. Additionally, **7** interacts with several other residues, such as Thr805, Val330, and Asn130, which are all important for the binding between cardiac glycosides and NKA. As a result, **7** exhibited strong binding affinity and substantial cytotoxicity (Table 1) [32]. These results indicate that the C-17 lactone unit is important but not necessary for digoxin to bind to NKA to mediate its antitumor activity. Any appropriate substituents at the C-17 position that could dock with the hydrophobic funnel of NKA may improve the binding between digoxin and NKA, and the resultant antitumor potential of this cardiac glycoside.

Both **8** and **9** have a benzylidene substituent at the C-21 position, but their binding poses, docking scores, and cytotoxicity are dramatically different. Compound **8** cannot bind to the cationic pocket of hNKA as deeply as **2** (Figure 3D and Figures S3 and S4) in any of its docking poses that belong mainly to cluster-I or cluster-II. Compared with **2**, the steroid core of **8** seems to adopt an opposite orientation to insert into the binding pocket, owing to its overall rigid structure and the large lactone unit. The α-surface of its steroid moiety interacts partially with the αM1–αM2 loop (124-EEP-126) and Leu801 in the αM5–αM6 loop in cluster-I, and with the whole αM1–αM2 loop (119-QAATEEEP-126) in cluster-II. All of these loops consist of residues with polar and negatively charged sidechains that are not supportive of interacting with the apolar α-surface of the steroid moiety of **8**. In addition, the carbonyl group of **8** in clusters I and II orients toward the Asn130 pocket, but this causes an undesirable effect on the binding between **8** and hNKA. Thus, **8** showed a low binding affinity and accordingly weak cancer cell cytotoxicity (Table 1) [33].

In contrast, the C-21 benzylidene unit of **9** is larger than that of **8**, which makes the lactone unit of **9** face downward in all of its binding poses. The conformations of this oriented lactone unit of **9** in the binding pocket of hNKA are similar, but these poses make it difficult for this compound to insert into the binding pocket of hNKA (Figure 3E and Figures S3G,H and S4B). However, the C-21 *N*,*N*-dimethylamine group could provide a polar interaction with the cation-binding site composed of several polar residues, including Glu787, Glu335, and Asp812, and the large lactone unit of **9** was found to fit into the Asn130 pocket very well. Additionally, the α-surface of its steroid core orients toward the hydrophobic surface of αM4–αM6. All of these aspects support **9** in being able to dock well to the hNKA binding cavity, and thus this compound showed a high binding affinity, with the docking scores being −11.8 (average)/−12.9 (minimal) kcal/mol (Table 1).

As discussed above, the Asn130 side docking pocket seems to play an important role in the interaction of a cardiac glycoside and NKA. Docking to the Asn130 pocket by the OH-12β group of digoxin (**2**) or the formation of a hydrogen bond between this hydroxy group and the Asn130 sidechain would increase the binding affinity of **2**, and such binding could be improved by increasing the size of its OH-12β group through introducing another hydrogen bond donor. The modified substituent may insert more deeply inside the Asn130 pocket, and the hydrogen bond formed could enhance the binding between the modified digoxin and hNKA. In addition, as mentioned for **9**, an appropriate substituent at the C-21 position, such as the *N*,*N*-dimethylaminobenzylidene moiety, can dock to the Asn130 pocket, and such a docking could strengthen the binding between a cardiac glycoside and hNKA. Therefore, when guided by docking to the Asn130 pocket, **2** could be modified synthetically by changing substituent at the C-12 position or by introducing an additional moiety at the C-21 position to improve its interaction with NKA and the resultant antitumor property.

### 2.4. Impact of the 3β-Glycosyl Group on the Binding between Digoxin and hNKA

In the crystal structure of the pig kidney NKA (α1β1γ) and digoxin (**2**) complex, the first 3β-attached glycosyl moiety was found to occupy a wide cavity of NKA, lined with polar residues at the αM1, αM2, αM4, αM7, and αM8 loops [10]. Thus, the absence of the C-3 saccharide moiety in digoxin, such as the aglycone, (+)-digoxigenin (**10**), and its dehydrated analogue, (+)-8(9)-β-anhydrodigoxigenin (**11**) (Figure 3A), could result in decreased binding affinities. As shown in Figure 3F and Figures S3I and S4C, compound **10** exhibits a binding pose similar to that of **2** in the hNKA pocket, but it loses the interaction between the first glycosyl group and the entrance of the cation binding pocket of hNKA observed for **2**. This loss causes a decreased binding affinity (Table 1), and thus this aglycone cannot bind to hNKA properly. In addition, **11** inserts into the binding cavity of hNKA with fairly uniform conformations. Removal of the OH-14β group results in its steroid core being rotated around 30 degrees toward the Phe791 sidechain, and its lactone unit flipped around 180 degrees to avoid the steric clash with the Val322 sidechain of hNKA. These changes cause its hydroxy groups to orient to the other side of the cavity to further decrease its binding to hNKA (Figure 3G and Figures S3I and S4C), and thus, **11** binds weakly to hNKA. Therefore, both **10** and **11** do not bind well to hNKA, and thus their bioactivities could not arise from targeting hNKA directly.

Using Autodock Vina, the docking scores were calculated for all of compounds **1**–**11**. Interestingly, these values were found to correlate well with their cancer cell cytotoxicity (Table 1 and Figure S5). For example, digitoxin (**1**) showed smaller docking scores (−11.8/−12.5 kcal/mol) and more potent cytotoxicity (IC_50_ < 0.5 μM toward human HT-29 colon and MDA-MB-231 breast cancer cells) than digoxin (**2**, docking scores of −11.6/−12.2 kcal/mol, IC_50_ < 2.2 μM toward HT-29 and MDA-MB-231 cells) (Table 1). Consistently, analogous values calculated from the binding between **5** and hNKA were greater than those from **3** and **4**, and this similar trend was also observed when comparing **8** with **9** (Table 1). However, the docking scores of both **10** and **11** seem not to correlate with their cytotoxicity, which is probably because neither compound targets hNKA. Thus, the docking scores could be used potentially in the prediction of cytotoxicity of cardiac glycosides if they exert their bioactivity by targeting NKA directly. Additionally, it is worth noting that compound **9** showed the smallest docking scores (−11.8/−12.9 kcal/mol) and the most potent cytotoxicity against HeLa cells (IC_50_ 0.26 μM) [23], suggesting that this compound could be further modified structurally for the development of NKA-targeted antitumor agents.

### 2.5. Binding between Digoxin or Its Aglycone and FIH-1 and NF-κB

It has been reported that digoxin targets not only NKA but also hypoxia-inducing factor 1α (HIF-1α) and nuclear factor kappa B (NF-κB) to mediate its antitumor potential [19,21]. Tissue hypoxia occurs commonly in solid tumors, and induction of HIFs mediates many proteomic and genomic changes to support tumor growth. Thus, HIF-1α has been well-documented for its important role in supporting metastasis and drug resistance in breast cancer, and inhibition of this protein has been regarded as an essential strategy in breast cancer treatment [35]. Digoxin was found to inhibit HIF-1α effectively to suppress tumor growth [36], and it also prohibited HIF-1α synthesis, vascular endothelial growth factor (VEGF), and N-Myc downregulated gene 1 (NDRG1) in A549 human lung cancer cells to show its cytotoxicity [37]; it suppressed HIF-1α and blood vessel formation in C4-2 castration-resistant prostate tumors [38]. However, the docking profile for digoxin and HIF-1α has not been reported thus far.

**Figure 4:**
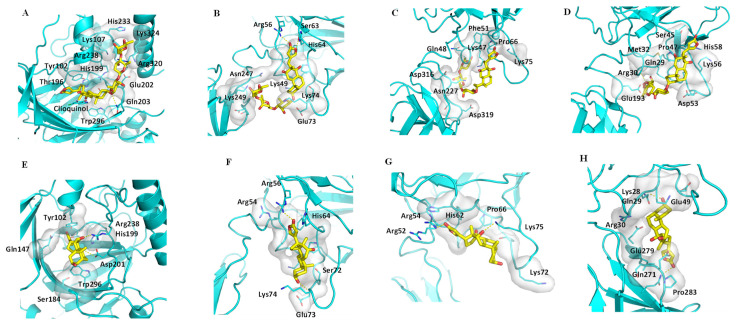
Docking profiles for digoxin (**2**, yellow) and FIH-1 (**A**) and the subunits p50 (**B**), p52 (**C**), and p65 (**D**) of NF-κB; and for digoxigenin (**10**, yellow) and FIH-1 (**E**) and the subunits p50 (**F**), p52 (**G**), and p65 (**H**) of NF-κB. Digoxin (**2**) can bind to FIH-1 and the subunits p50 and p65 of NF-κB, but digoxigenin (**10**) cannot.

Factor inhibiting HIF-1 (FIH-1) is a protein that interacts with HIF-1α to inhibit its transcriptional activity [39], and it (PDB ID: 3KCX) contains a central cavity for the substrate and inhibitors to dock. The crystal structure of complex of FIH-1 and clioquinol, a FIH-1 inhibitor (Figure S6), showed that substituents at the C-7 and C-6 positions of clioquinol orient toward the open space of FIH-1 to allow additional ligands to bind, and filling this space could improve the binding affinity of clioquinol [40]. Docking profiles for digoxin (**2**) and its aglycone, digoxigenin (**10**), and FIH-1, showed that **2** interacts with the Arg238, His199, Tyr102, Thr196, Trp296, Gln203, and Glu202 sidechains of FIH-1 (Figure 4A). The lactone unit of **2** occupies the central cavity of FIH-1, where the steroid core interacts with the hydrophobic resides, and a hydrogen bond is formed between oxygen at the C-3 position of **2** and the Gln203 sidechain of FIH-1. Such a binding mode is also supported by interaction of the saccharide moiety of **2** and the Glu202, Arg320, Lys324, and Lys107 sidechains of FIH-1 (Figure 4A). Thus, both the steroid core and the C-3 glycosyl group of digoxin could insert into the open space of FIH-1 to support the docking of its lactone unit to the central cavity of FIH-1. Thus, **2** showed a great docking score value (−10.6 kcal/mol) from its binding to FIH-1 (Table 2). However, the lack of a saccharide moiety of **10** results in partial loss of binding affinity (docking score −9.2 kcal/mol) (Table 2), and the whole molecule may rotate in the cavity. These de-potentiated greatly the binding between **10** and FIH-1 (Figure 4E), even though the aglycone occupies the cavity at the similar position to that for **2** (Figure S7). Therefore, **2** binds to FIH-1 strongly, but **10** does not.

NF-κB plays a key role in inflammation and human pathobiology, and inhibition of this protein could support the treatment of autoimmune and lymphoproliferative disorders [41]. Previously, digoxin was found to inhibit the activation of the NF-κB pathway in Raji human Burkitt’s lymphoma cells, indicating that this cardiac glycoside may target NF-κB to mediate its antitumor activity [42]. To test this hypothesis, digoxin (**2**) and digoxigenin (**10**) were docked into three subunits of NF-κB, including the p50 (PDB ID: 1NFK), the p52 (PDB ID: 1A3Q), and the p65 (PDB ID: 2RAM) subunits. Digoxin binds well to the p50 DNA binding pocket, with the steroid moiety occupying the groove formed by the Gly52-Arg56 and Ser63-Lys74 loops, which have some hydrophobic residues at the bottom, such as Phe53, Leu67, Ala70, and several glycines. Furthermore, the lactone unit of **2** could form hydrogen bonds with Arg56 and Ser63, which further supports the overall interaction. Additionally, the saccharide moiety of **2** could reach other parts of the p50 subunit of NF-κB that cross the DNA binding pocket, which would enhance the binding affinity of digoxin and the p50 subunit (Figure 4B). Differentially, **10** lies down in the cavity. The lactone unit of **10** interacts with Phe51 by pi-pi stacking, and two hydrogen bonds are formed between the lactone carbonyl and the C-3 hydroxy group of **10** and the Arg56 and Glu73 sidechains of the p50 subunit, respectively. However, the steroid core of **10** would not interact greatly with the cavity of the p50 subunit, which has a hydrophobic bottom and a charged and polar edge (Figure 4F), and thus **10** does not bind well to this subunit of NF-κB.

The Glu58–Gly64 part in the binding pocket of the subunit p52 is larger than that the equivalent Glu60–Gly66 part in the p50 subunit, and the sidechain of His62 moves far away from the ligand binding site. As a result, the lactone unit of **2** could not insert deeply to the end of the pocket to form a hydrogen bond. In addition, the steroid core of **2** would not interact strongly with the bottom of the binding groove, even though two hydrogen bonds are formed between its glycosyl group and the Gln48 and Lys47 sidechains of the p52 subunit, which provides some ligand–protein interaction (Figure 4C). Similarly, **10** adopts the same binding pose as that of **2**, which would not support any effective binding for it, and its steroid core could not interact strongly with the cavity of the p52 subunit (Figure 4G). Therefore, neither **2** nor **10** binds well with the p52 subunit of NF-κB.

In the p65 subunit, the binding pose of **2** is similar to that of p52, but the whole structure can bind inside the pocket. In addition, its steroid moiety interacts with the hydrophobic bottom of the binding pocket, which further strengthens the binding between digoxin and the p65 subunit of NF-κB (Figure 4D). However, **10** was only able to fit into different sites in the pocket when compared with **2**, which did not support its binding to this subunit (Figure 4H), and thus **10** does not bind to the p65 subunit. Therefore, **2** binds to the p50 and p65 subunits (docking score < −8.5 kcal/mol) (Table 2) but not the p52 subunit of NF-κB, whereas **10** binds to none of these proteins (Figure S7) (docking score > −7.7 kcal/mol) (Table 2).

### 2.6. Binding between Digoxin or Its Aglycone and HDAC and IAP

**Figure 5:**
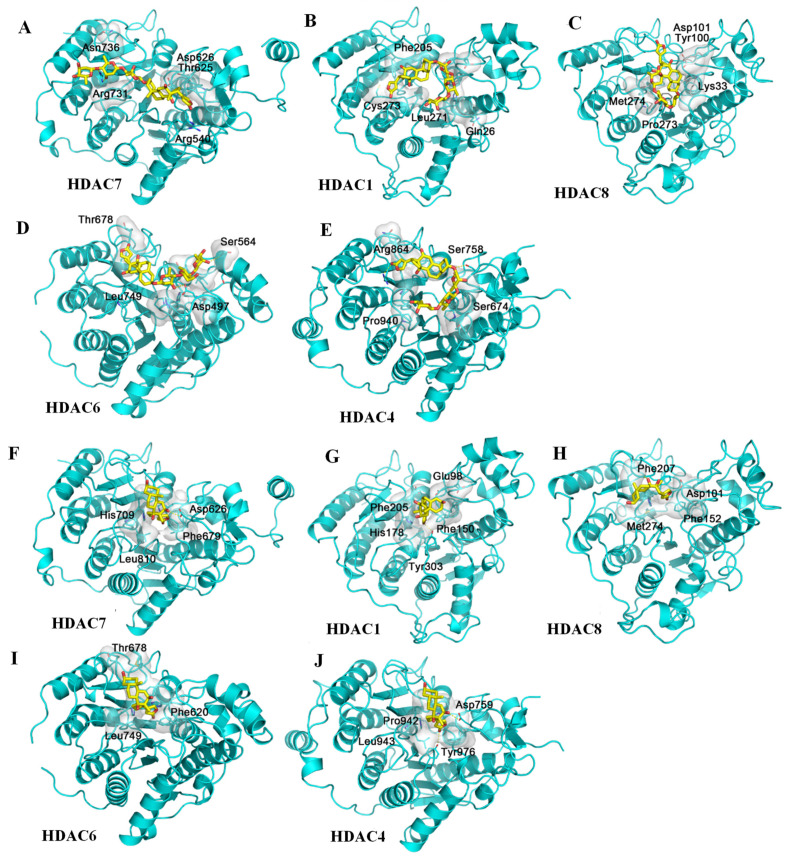
Docking profiles for digoxin (yellow) and histone deacetylases HDAC7 (**A**), HDAC1 (**B**), HDAC8 (**C**), HDAC6 (**D**), and HDAC4 (**E**); and digoxigenin (yellow) and HDAC7 (**F**), HDAC1 (**G**), HDAC8 (**H**), HDAC6 (**I**), and HDAC4 (**J**). The HDAC family has three classes and over 10 members in humans, but the structures of these proteins are notably similar, with the same binding site being at the center of each HDAC domain. Five crystal structures of different HDACs were obtained as the receptors for the docking of digoxin and digoxigenin. The center pocket of HDACs is too small for the steroid motif and the lactone unit of digoxin to be inserted, and thus, digoxin could not bind to HDACs (**A**–**E**), even though two different sizes of docking boxes were used to carry out docking. However, the docking profiles for digoxigenin and HDACs are greatly improved (**F**–**J**) when compared with those for digoxin.

Besides binding to its molecular targets, digoxin was also found to mediate its antitumor activity by induction of apoptosis and autophagy [36,42,43,44,45], for which both histone deacetylases (HDACs) and inhibitor of apoptosis (IAP) have been proposed as important targets [46,47,48]. There are three classes with more than 10 members in the human HDAC family, with the similarity of the members in the same class being higher than that in different classes; the structures of these proteins are notably similar (Figure S8). Thus, five crystal structures from different HDAC groups were selected as the receptors for docking with digoxin (**2**) and digoxigenin (**10**), with two different sizes of docking box being used. These variants include HDAC7 (PDB ID: 3C10), HDAC1 (PDB ID: 4BKX), HDAC8 (PDB ID: 5DC8), HDAC6 (PDB ID: 5EDU), and HDAC4 (PDB ID: 5ZOO), which all show similar structures and functions. The docking profiles showed that the central binding pocket of HDACs is too small to accommodate **2**, indicating weak binding affinity for these proteins (docking score > −7.0 kcal/mol) (Table 2). However, compared with **2**, the binding between digoxigenin (**10**) and the HDACs is stronger (docking score < −7.5 kcal/mol) (Table 2), even though both binding poses, with or without the lactone unit of **10** inserting into the central cavity, could not reach the binding pocket deeply (Figure 5 and Figures S9 and S10).

**Figure 6:**
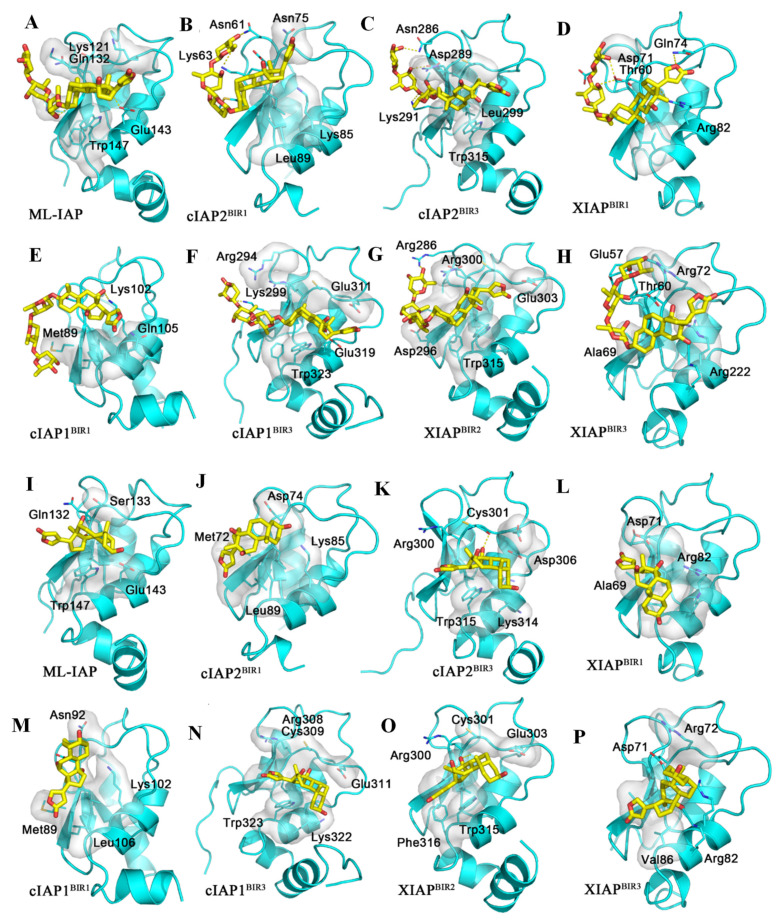
Docking profiles for digoxin or digoxigenin and inhibitor of apoptosis (IAP). The IAP family includes X chromosome-linked IAP (XIAP), cellular IAP-1 (cIAP1), cellular IAP-2 (cIAP2), and melanoma IAP (ML-IAP). The active domains of the IAP proteins are the baculoviral IAP repeat (BIR) domains, and most of these proteins have more than one BIR domain, for which the sequence alignment was generated by Clustal X, and sequences and structures of the active sites of the BIR domains are notably similar. A total of eight BIR domain structures were obtained from the PDB database, belonging to different IAP proteins, to which digoxin and digoxigenin were docked by Autodock Vina. Shown above are the docking profiles for digoxin (yellow) and ML-IAP (**A**), cIAP2^BIR1^ (**B**), cIAP2^BIR3^ (**C**), XIAP^BIR1^ (**D**), cIAP1^BIR1^ (**E**), cIAP1^BIR3^ (**F**), XIAP^BIR2^ (**G**), and XIAP^BIR3^ (**H**); and digoxigenin (yellow) and ML-IAP (**I**), cIAP2^BIR1^ (**J**), cIAP2^BIR3^ (**K**), XIAP^BIR1^ (**L**), cIAP1^BIR1^ (**M**), cIAP1^BIR3^ (**N**), XIAP^BIR2^ (**O**), and XIAP^BIR3^ (**P**). When using 4KMN as the receptor, digoxin and digoxigenin (yellow) lie on the surface of the binding site, and the steroid motif fits into one of the grooves. The hydrophobic residues at the bottom of the pocket of IAPs may provide some beneficial interactions of these two compounds. However, their overall conformation limits the interaction with the other groove, and the lack of any hydrogen bond interactions makes the bonding between these compounds and IAPs very weak (**A**–**P**). Thus, both compounds do not bind to IAPs well.

The inhibitor of apoptosis (IAP) family includes X chromosome-linked IAP (XIAP), cellular IAP-1 (cIAP1) and IAP-2 (cIAP2), and melanoma IAP (ML-IAP). The active domains of these proteins are baculoviral IAP repeat (BIR) regions; most of them have more than one BIR domain. The sequence alignment of these BIR domains showed that the sequences and structures of the active site of the BIR domains are similar (Figure S11), but the binding pocket of BIR, as shown in the inhibitor (Q27454369) co-crystal structure of cIAP1 (4KMN), is quite flat and shallow (Figures S12 and S13). Two small grooves are formed by hydrophobic residues in the bottom and charged residues at the wall for the cIAP1 inhibitors to insert. Digoxin (**2**) was docked to different BIR domains from several members of the IAP family, including ML-IAP (PDB ID: 2I3H), XIAP (PDB ID: 2POI, 6GJW, 3UW4), cIAP1 (PDB ID: 3M1D, 4KMN), and cIAP2 (PDB ID: 3M0A, 2UVL), and it could bind to all these IAPs. However, compound **2** occupies the surface of the binding site, with the steroid motif fitting into one of the grooves, but it could not reach both grooves, even with support from the hydrophobic residues of cIAP1 (Figure S14). In addition, the conformation of this cardiac glycoside limits its interaction with cIAP1, and the lack of hydrogen bond interaction attenuates the binding affinity of digoxin and cIAP1. Similarly, the rigid steroid motif of digoxigenin (**10**) is not suitable for the shallow and flat surface of the binding sites, which prevents it from reaching the grooves of cIAP1 or forming hydrogen bonds with the proteins (Figure 6). Therefore, neither **2** nor **10** could interact strongly with cIAP1. Similar binding situations were observed for both **2** and **10** and other IAPs, including ML-IAP, XIAP, and cIAP2 (Figure 6) (docking score > −7.0 kcal/mol) (Table 2).

The low binding affinities indicate that digoxin does not induce apoptosis and autophagy in human cancer cells by targeting HDACs or IAPs directly, but it may mediate these activities through the HIF-1α, NKA, and NF-κB signaling pathways [36,42,43,44,45]. However, better binding between digoxigenin and HDACs was observed, which suggests that this aglycone may exhibit potential antitumor activity through induction of the HDAC-mediated autophagy or signaling.

### 2.7. Binding between Digoxin or Its Aglycone and PI3K

**Figure 7:**
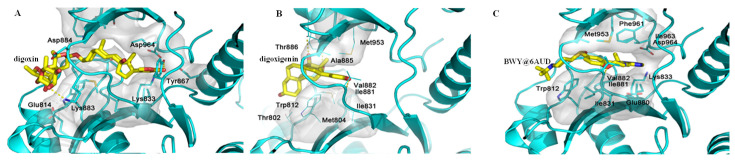
Docking profiles for digoxin, digoxigenin, and BWY, a phosphoinositide 3-kinase (PI3K) inhibitor, and PI3K. The rigid steroid core and the long glycosyl group of digoxin (yellow) prohibit it from fitting into the pocket of PI3K, and thus this cardiac glycoside does not bind to PI3K (**A**). However, the steroid motif of digoxigenin (yellow) may form a pi–pi interaction with Tyr812, and its hydroxy groups at the C-12 and C-14 positions form hydrogen bonds with the sidechains of PI3K. Additionally, the lactone unit of digoxigenin would interact with the entrance of pocket of PI3K, and thus this aglycone could interact with PI3K (**B**). Strong binding was observed for BWY (yellow) and PI3K (**C**).

NKA transports potassium ions in and sodium ions out, and Na^+^ was found to be required in the extracellular solutions to drive glucose transport, indicating a correlation between the sugar and sodium pumps. However, the NKA-inhibitory digoxin was not found to inhibit glucose uptake, suggesting that digoxin-mediated cancer cell cytotoxicity does not result from inhibiting glucose transport [19]. Phosphoinositide 3-kinase (PI3K) is an important protein for tumor cell biological activities, and inhibition of the PI3K pathway can yield multifaceted tumor cell-extrinsic effects to support cancer treatment [49]. In addition, PI3K signaling is associated with the regulation of several metabolic effects through its activity on Akt and mTOR, as supported by a shift towards a highly glycolytic phenotype observed in cancer cells by PIK3CA mutations [50]. To test its potential impact on PI3K, digoxin was docked into the active pocket of the PI3K crystal structure (PDB ID: 6AUD), which is formed by the Ile828-Leu838, Leu864-Thr872, and Phe961-Leu969 sidechains for BWY, a PI3K inhibitor (Figure S15). The docking profile showed that the rigid steroid core and the long saccharide moiety of digoxin prohibited it from fitting into the pocket, and hence, digoxin could not bind to PI3K. Differentially, a pi–pi interaction was observed between **10** and Tyr812 of PI3K, and hydrogen bonds were formed between the hydroxy groups connected at the C-12 and C-14 positions of **10** and PI3K (Figure 7 and Figure S16). Thus, **10** could bind to PI3K.

## 3. Discussion

It has been well documented that the antitumor potential of cardiac glycosides correlates with their inhibition of NKA, which could result from their binding to this target [18,19,20]. In the present investigation, the docking scores calculated for digoxin and several of its synthetic derivatives were found to correlate with their cancer cell cytotoxicity, and thus, these values may be used to guide future synthetic modifications of cardiac glycosides. This also indicates the potential of molecular modeling in the virtual screening of bioactive cardiac glycosides or other natural products to support or supplement bioassays.

The Asn130 side docking pocket of NKA revealed in the present study seems to be an important target for the discovery of novel bioactive digoxin derivatives. Based on this insight, digoxin could be modified to improve its antitumor activity, and its C-12 and C-21 positions may be promising targets in this regard. Additionally, this Asn130 pocket may be used in investigations of other NKA inhibitors.

One of the serious challenges for the development of digoxin (**2**) as a new anticancer drug is its narrow clinical therapeutic index, which potentially results from its inhibition of NKA [19]. Fortunately, digoxin was found to inhibit HIF-1α and the activation of the NF-κB pathway effectively to exhibit its antitumor activity [36,37,38,42], which has been supported by the present investigation. Digoxin binds to FIH-1 (factor inhibiting HIF-1) and the p50 and p65 subunits of NF-κB, and thus it could directly target these proteins to mediate its antitumor potential. Based on these results, some novel anticancer agents could be discovered by modifying digoxin to target HIF-1α and NF-κB, to overcome any challenges derived from an interaction with NKA.

Digoxigenin (**10**) has been shown to have inhibitory activity against NKA [17] and was reported recently to interact strongly with this protein by molecular docking [51]. However, in the present investigation, this steroidal molecule was found to bind to HDAC and PI3K rather than NKA. These observations could have resulted from the different molecular modeling methodologies used, but nonetheless indicate that digoxigenin (**10**) may inhibit NKA by direct action or through a signaling transduction mechanism. In addition, both HDAC and PI3K regulate tumor proliferation, autophagy, and metabolism [46,49,50]; and the antitumor activity of the cardiac glycoside, cerberin, has been reported to be mediated through the PI3K/Akt/mTOR signaling pathway [52]. Thus, the present results suggest that digoxin (**2**) and its aglycone **10** mediate their antitumor activity through different mechanisms of action. Furthermore, structural modifications of **10** could generate new compounds that target HDAC and PI3K to treat cancer effectively.

Previous laboratory research work by us and other groups has shown that digoxin induces cancer cell apoptosis, and it targets not only NKA but also HIF-1α and NF-κB to mediate its antitumor potential [19,36,37,38,42]. Additionally, the sodium pump was evidenced to correlate with the sugar pump [19]. However, to the best of our knowledge, the docking profiles for digoxin and these molecular targets have not been investigated. The present investigation indicates that digoxin binds to FIH-1, NKA, and NF-κB, but digoxigenin docks to the binding cavities of HDACs and PI3K, suggesting that digoxin directly targets FIH-1, NKA, and NF-κB to mediate its antitumor potential, but digoxigenin may exhibit such activity through the HDAC and PI3K signaling pathways.

## 4. Materials and Methods

### 4.1. Compounds and Biological Evaluation

Compounds **1** and **2** were purchased from Sigma-Aldrich (St. Louis, MO, USA), and compounds **3**–**6**, **10**, and **11** were prepared in the previous work by our group; the cancer cell cytotoxicity and molecular targets were already reported [19,31]. In turn, compounds **7**–**9** were selected from the literature, which also reported their bioactivities [23,32,33].

### 4.2. Sequence Alignment

The sequence of the hNKA (length: 1023) was obtained from UniProt database, and the crystal structures of sNKA (length: 1021)] were selected from Protein Data Bank (PDB). The sequence alignment for hNKA and sNKA was generated by Clustal X version 2.0 [26] and further refined manually, from which the amino acid identity was found to be up to 98%, and all local areas of structural and functional importance were well conserved.

### 4.3. Molecular Modeling for hNKA

Using crystal structures of 3N23 (the complex of NKA E2P-ouabain), 4HYT (the complex of NKA E2P-ouabain with bound Mg^2+^), 4RES (the complex of NKA E2P-bufalin with bound K^+^), and 4RET (the complex of NKA E2P-digoxin with bound Mg^2+^) from the PDB database as the reference, a total of 100 structures were produced for the homology model by Modeller 9.10 [53]. Three different criteria were used to evaluate and filter results, including discrete optimized protein energy (DOPE) and molecular probability density function (molpdf) scores and Ramachandran plot. Based on DOPE and molpdf scores, the best model was selected, and the target model was evaluated by investigating the psi/phi Ramachandran plot using the RAMPAGE server (http://mordred.bioc.cam.ac.uk/-rapper/rampage.php, accessed on 10 March 2021). The modeling structure was refined with a 20 ns MD simulation by Gromacs 2019.4.

### 4.4. Docking Simulation for NKA

The modeling structure of hNKA was used as the receptor. The 3D structures of **3**–**11** were built in Maestro and prepared by LigPrep from Schrödinger Suite 2018-2 (Schrödinger Suite 2018-2 Protein Preparation Wizard (Schrödinger, LLC, New York, NY, USA, 2018)). The geometric optimization was performed using the OPLS3 (optimized potentials for liquid simulation 3) force field with all possible ionization states at pH 7.4 ± 0.1 created by Epik, a software program for pKa prediction and protonation state generation [54]. The conformations of **3**–**11** generated by LigPrep were used for the molecular docking against the receptor by Autodock Vina [55,56]. A rectangular box (25 × 25 × 30 Å^3^) centered around the center of Leu800, Ile794, Glu124, and Gln118 (residue index in hATPase, close to oxygen at the C-3 position of digoxin in crystal structure 4RET) defines the region that the ligands can explore, which could cover nearly all of α-M1−M6 of hNKA. Digoxin (**2**) was also docked to the receptor as the reference.

### 4.5. Docking Profiles for ***2*** or ***10*** and HDAC

Autodock Vina was used to generate the docking profiles [55], and MGLTools 1.5.6 [57] was used to set up the docking box. Rectangular boxes (30 × 25 × 30 Å^3^) with their centers at the centers of C-terminals of the α1 helix and β6 sheet were set up in the active pockets of HDACs. There are three classes with more than ten members in the human histone deacetylase (HDAC) family, with the members in the same class being more similar than those in different classes. The structures of the proteins are notably similar to each other. Five crystal structures from different HDACs were obtained as the receptors for the docking with digoxin or digoxigenin.

### 4.6. Docking Profiles for ***2*** or ***10*** and FIH-1

Autodock Vina was used to generate the docking profiles for **2** or **10** and FIH-1 [55], and MGLTools 1.5.6 [57] was used to set up the docking box, and rectangular boxes (30 × 20 × 30 Å^3^) with centers at the center of two sheet domains of FIH-1′s structure being used for docking research.

### 4.7. Docking Profiles for ***2*** or ***10*** and IAP

Autodock Vina was used to generate the docking profiles for **2** or **10** and IAPs [55], and MGLTools 1.5.6 [57] was used to set up the docking box. Inhibitor of apoptosis (IAP) family including X chromosome-linked IAP (XIAP), cellular IAP-1 (cIAP1), cellular IAP-2 (cIAP2), and melanoma IAP (ML-IAP). The active domain of IAP proteins is a baculoviral IAP repeat (BIR) domain, and most of these proteins have more than one BIR domain. The active sites of the BIR domains are notably similar in both sequence and structure. A total of eight BIR domain structures were obtained from PDB database, which belong to different IAP proteins. The inhibitor co-crystal structure of cIAP1 (4KMN) was used as the reference to investigate the binding between small molecules and IAPs. All of the structures were superimposed to 4KMN, and rectangular boxes (22 × 15 × 15 Å^3^) centered on the ligand of 4KMN were set.

### 4.8. Docking Profiles for ***2*** or ***10*** and NF-κB

Autodock Vina was used to generate the docking profiles for **2** or **10** and NF-κB [55], and MGLTools 1.5.6 [57] was used to set up the docking box. Digoxin or digoxigenin was docked into three different the subunits of NF-κB complex, including p50 (PDB ID: 1NFK), p52 (PDB ID: 1A3Q), and p65 (PDB ID: 2RAM). All of the structures were superimposed to p50 structure, and rectangular boxes (20 × 26 × 20 Å^3^) with centers close to the center of the DNA structure in 1NFK were set.

### 4.9. Docking Profiles for ***2*** or ***10*** and PI3K

Autodock Vina was used to generate the docking profiles for **2** or **10** and PI3K [55], and MGLTools 1.5.6 [57] was used to set up the docking box. PI3K crystal structure (PDB ID: 6AUD) was used for docking, and a rectangular boxes (20 × 20 × 30 Å^3^) with the center at the center of co-crystal ligand of PI3K structure (PDB ID: 6AUD) were set. Digoxin or digoxigenin was docked into the active pocket of PI3K crystal structure (PDB ID: 6AUD). The co-crystal structure of PI3K in complex with an inhibitor, BWY (PDB ID 6AUD) [58], shows strong interactions between the ligand and the ATP binding site, formed by Ile828-Leu838, Leu864-Thr872, and Phe961-Leu969, and it was used to investigate the binding between **2** and **10**, and PI3K, respectively.

## 5. Conclusions

The present docking study confirmed that digoxin binds to NKA to mediate its antitumor potential; and the conformation and hydroxy group substitutions of its steroid core, the C-17 unsaturated lactone unit, and the C-3 saccharide moiety all play important roles in the mediation of its cancer cell cytotoxicity and in its interaction with NKA. The compound docking scores correlate well with their cytotoxicity; the additional Asn130 side pocket was found to play an important role. Additionally, digoxin binds to FIH-1 and NF-κB, but its aglycone, digoxigenin (**10**), docks to the active cavities of HDAC and PI3K, indicating that these two compounds mediate their antitumor potentials by interacting directly with these targets. Both HIF-1α and PI3K are major components of the tumor microenvironment (TME), which has been proposed as an essential target for cancer immunotherapy and for overcoming multidrug resistance and cancer relapse problems [59]. Therefore, both **2** and **10** can be regarded as lead compounds for the development of TME-targeted chemotherapeutic agents.

## Figures and Tables

**Table 1:** Docking score from the binding to NKA (PDB:4RET) and cytotoxicity (IC_50_ µM) of digitoxin and digoxin and its derivatives (**1**–**11**).

Compd.	Docking Score (kcal/mol)		Cytotoxicity	Compd.	Docking Score (kcal/mol)		Cytotoxicity
	Average	Minimal			Average	Minimal	
1	−11.8	−12.5	0.016 *^a^* 0.48 *^b^*	7	−10.8	−11.7	0.17 *^b^*
2	−11.7	−12.0	0.28 *^a^* 0.31 *^b^* 2.2 *^c^*	8	−10.7	−11.9	56 *^c^*
3	−10.9	−11.6	5.1 *^a^*	9	−11.8	−12.9	0.26 *^c^*
4	−11.1	−11.9	12 *^a^*	10	−9.5	−10.0	3.6 *^a^*
5	−10.3	−11.1	25 *^a^*	11	−9.8	−10.6	>50 *^a^*
6	−10.8	−11.3	>30 *^a^*				

Human *^a^* HT-29 colon [19], *^b^* MDA-MB-231 breast [32], and *^c^* HeLa cervical cancer cells [23,33].

**Table 2:** Docking scores (minimal, kcal/mol) of **1** and **10** from their binding to FIH-1, NF-κB, HDACs, IAPs, and PI3K.

Compd./Protein	1	10	Compd./Protein	1	10	Compd./Protein	1	10
FIH-1	−10.6	−9.2	HDAC8	−6.1	−8.3	XIAP^BIR1^	−6.0	−5.2
NF-κB (p50)	−9.2	−7.7	HDAC6	−7.0	−8.8	cIAP1^BIR1^	−5.8	−6.2
NF-κB (p52)	−8.5	−6.7	HDAC4	−5.3	−7.6	cIAP1^BIR3^	−5.7	−5.8
NF-κB (p65)	−8.8	−7.1	ML-IAP	−6.5	−6.3	XIAP^BIR2^	−6.1	−5.9
HDAC7	−6.9	−7.9	cIAP2^BIR1^	−5.9	−5.4	XIAP^BIR3^	−6.6	−5.2
HDAC1	−5.6	−8.3	cIAP2^BIR3^	−6.9	−6.1	PI3K	−6.4	−7.2

## Data Availability

The data presented in this study are available on request from the corresponding author, but they are not publicly available due to the requirements of ongoing research.

## Supplementary Materials

The following are available online. Molecular docking for **1**–**11** (Figures S1–S16) and the Ramachandran plot statistics for the hNKA model (Table S1).

## Sample Availability

Samples of the compounds **6**–**9** and **11** are not available from the authors.
