# Supplementary material for: Structural Insights into the Interactions of Digoxin and Na+/K+-ATPase and Other Targets for the Inhibition of Cancer Cell Proliferation
Source: Molecules. 2021 Jun 16;26(12):3672. doi: 10.3390/molecules26123672 (PMC8234910; doi:10.3390/molecules26123672)
Supplement: Supplementary file 1 [file molecules-26-03672-s001.zip › molecules-1240409-supplementary.pdf]

Supplementary Materials

Structural Insights into the Interactions of Digoxin and Na^+/K^+-ATPase and Other Targets for the Inhibition of Cancer Cell Proliferation

Yulin Ren[1, ‖,*], Sijin Wu[1,‖,#], Joanna E. Burdette[2], Xiaolin Cheng[1], and A. Douglas Kinghorn[1,*]

[1]Division of Medicinal Chemistry and Pharmacognosy, College of Pharmacy, The Ohio State University, Columbus, OH 43210; wusj@dicp.ac.cn (S.W.); cheng.1302@osu.edu (X.C.)
[2]Department of Pharmaceutical Sciences, College of Pharmacy, University of Illinois at Chicago, Chicago, IL 60612, United States; joannab@uic.edu (J.B.)
[#]Currently address: Laboratory of Molecular Modeling and Design, State Key Laboratory of Molecular Reaction Dynamics, Dalian Institute of Chemical Physics, Chinese Academy of Sciences, Dalian 116024, Liaoning Province, China
[‖]Equal contributions.
*Correspondence: ren.41@osu.edu (Y.R.); kinghorn.4@osu.edu (A.D.K.)

Table S1. Ramachandran plot statistics for the hNKA model

Description	Value	Percentage
Residues in the most favored regions [A. B. L.]	770	87.8%
Residues in the allowed regions [a. b. l. p.]	106	12.1%
Residues in the generally allowed regions [~a. ~b. ~l. ~p.]	1	0.1%
Residues in disallowed regions	0	0.0%
Number of non-glycine and non-proline residues	877	100.0%
Number of end-residues (excl, Gly, and Pro)	2	
Number of glycine residues (shown as triangles)	70	
Number of proline residues	43	
Total number of residues	992	

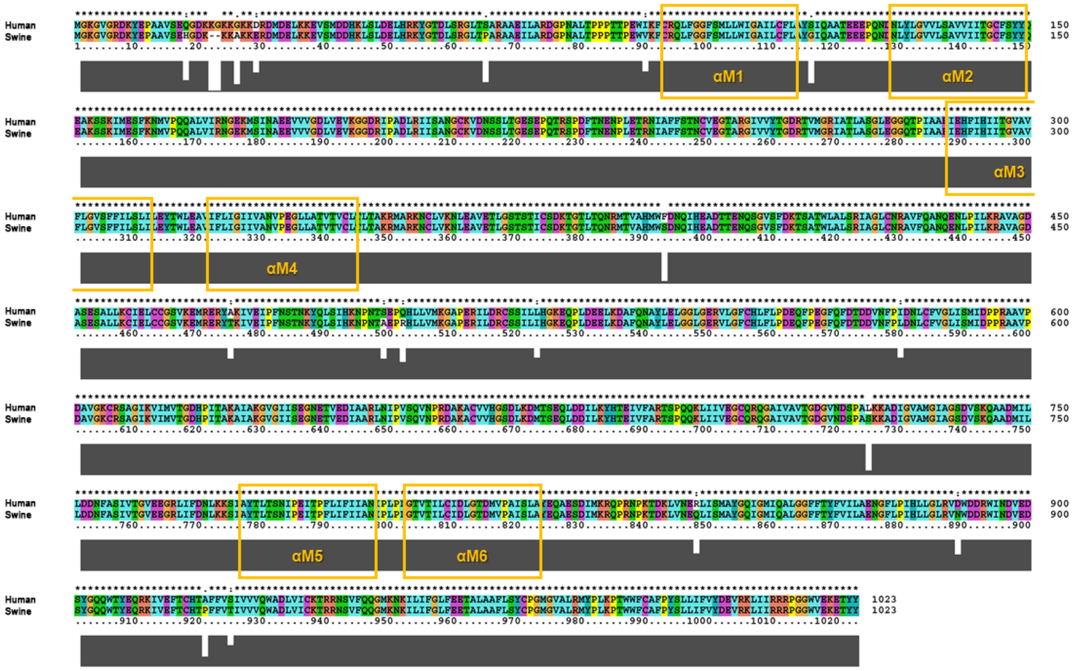

Figure S1. Sequence alignment between human and *Sus scrofa* Na^+/K^+-ATPases (hNKA and sNKA, respectively). Sequence alignment was generated by Clustal X version 2.0., used for hNKA modeling, and colored according to the chemical properties of the residues. Light yellow, aliphatic (P); cyan, polar uncharged (A, F, I, L, M, P, V, and W); yellow, aromatic (P); orange, Glycine (G); red, acidic (K and R); blue, basic (Y); pink, exceptional (C, D, and E); and green, positively charged polar (N, Q, S, and T). The black stripes indicate the conserved residues between two proteins, of which the height indicates the degree of similarity between hNKA and sNKA.

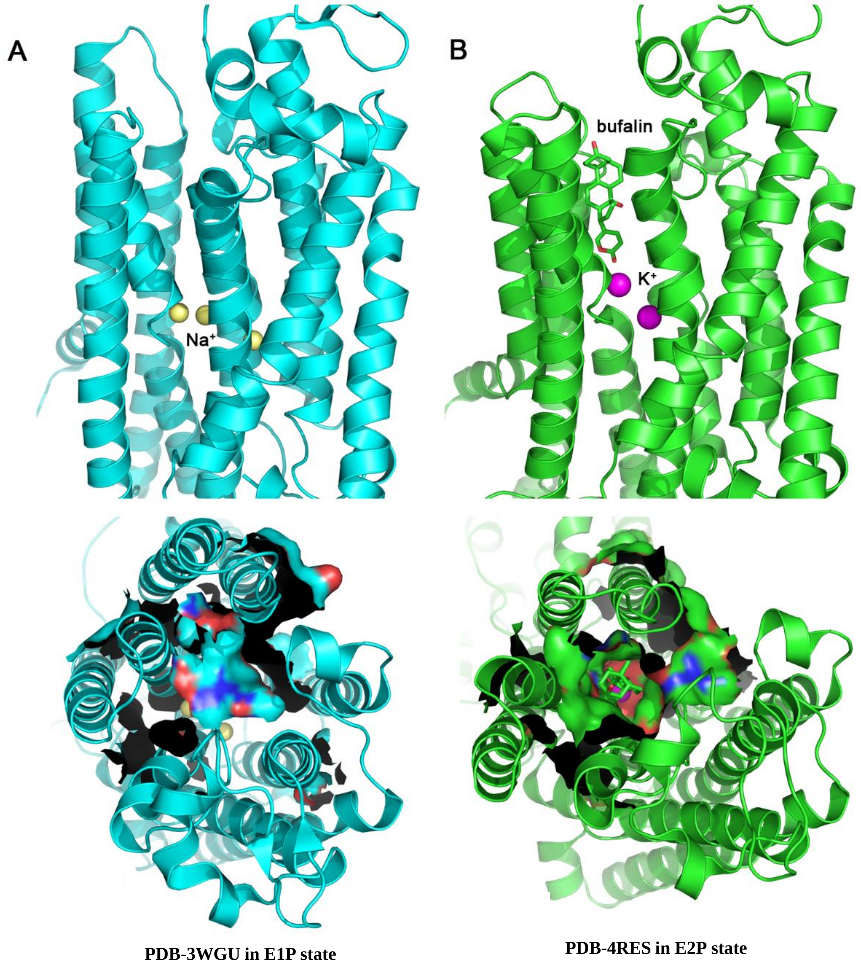

Figure S2. General overview for structures of Na^+/K^+-ATPase in E1P and E2P states. Column A (above and below). Structure of PDB-3WGU in the E1P state. Column B (above and below). Structure of PDB-4RES in the E2P state. The crystal structures of PDB 3WGU and PDB 4RES were obtained from PDB, and their secondary structures were generated by DSSP using PyMOL Version 2.0 (PyMOL v2).

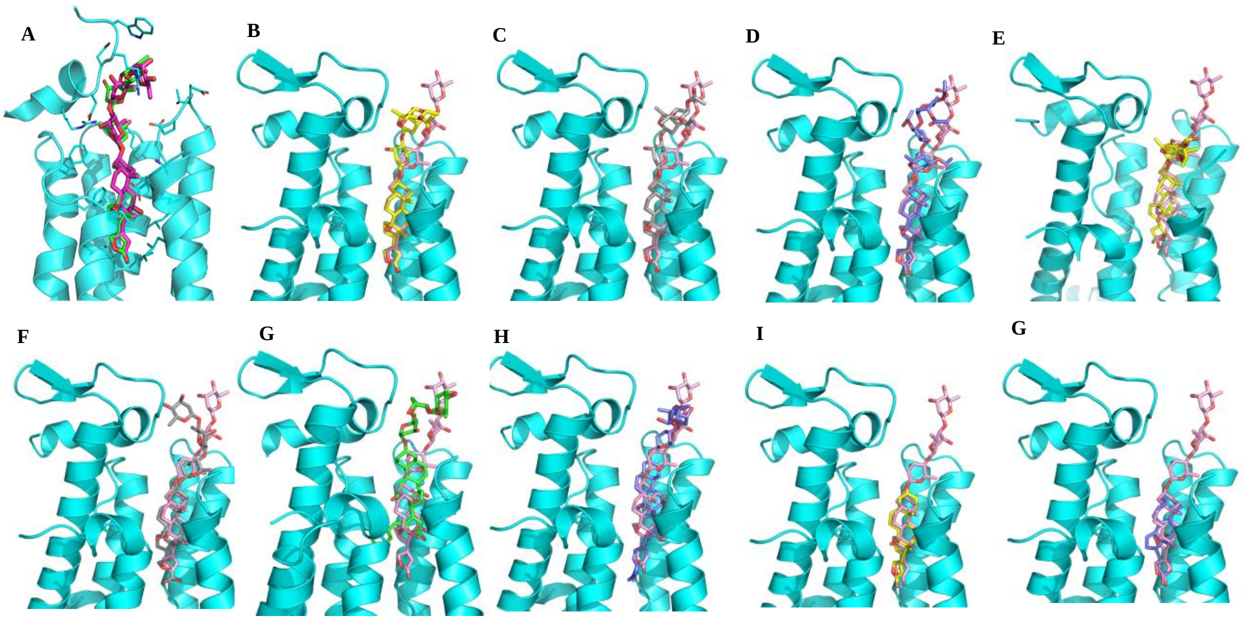

Figure S3. Overlaid docking profiles for digitoxin (**1**) and digoxin (**2**) and its derivatives **3**–**11** and hNKA [A. **1** (green) and **2** (pink); B. **2** (pink) and **3** (yellow); C. **2** (pink) and **4** (gray); D. **2** (pink) and **5** (blue); E. **2** (pink) and **6** (yellow); F. **2** (pink) and **7** (gray); G. **2** (pink) and **8** (green); H. **2** (pink) and **9** (blue); I. **2** (pink), **10** (yellow); J. **2** (pink) and **11** (blue).

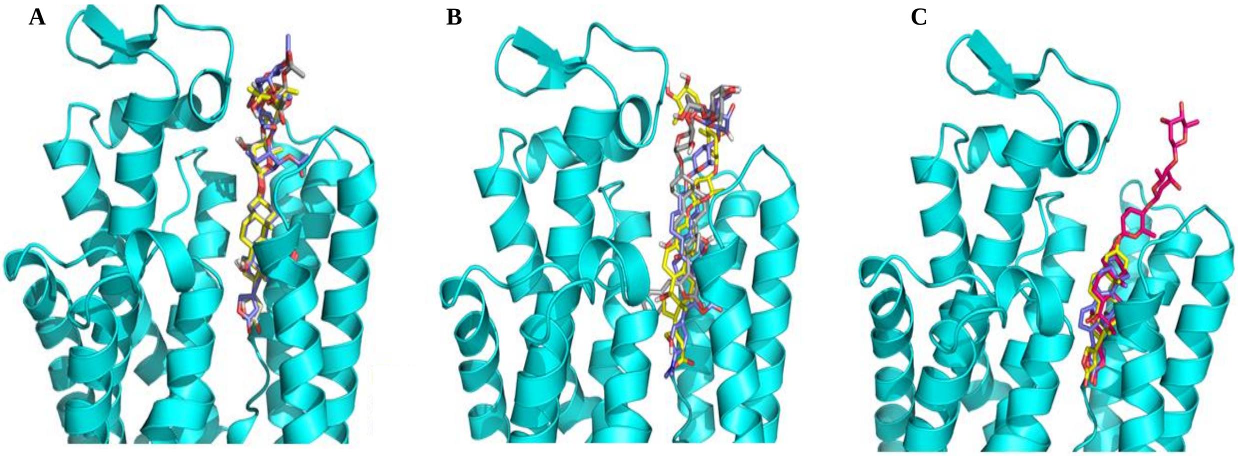

Figure S4. Overlaid docking profiles for digoxin (**2**) and its derivatives **3**–**5** and **7**–**11** and hNKA [A. **3** (yellow), **4** (gray), and **5** (blue). B. **7** (yellow), **8** (gray), and **9** (blue). C. **2** (pink), **10** (yellow), and **11** (blue).

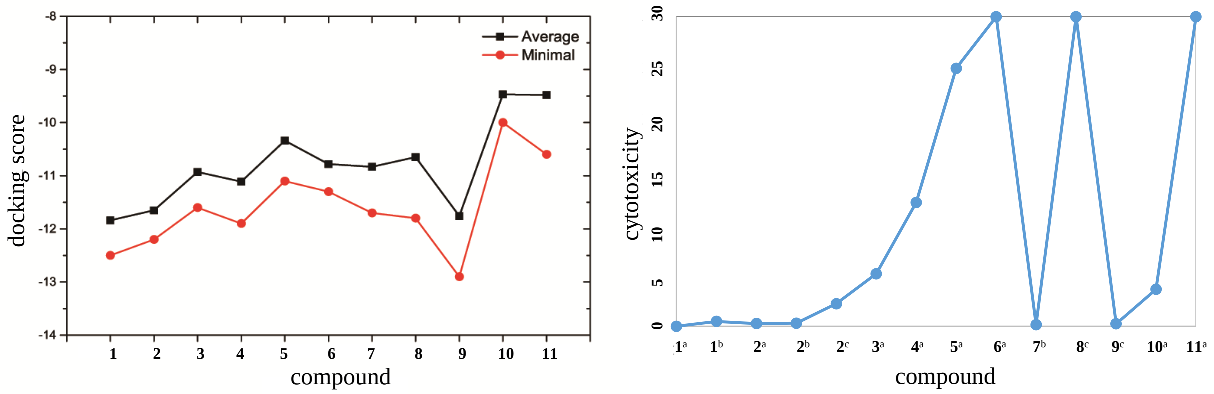

Figure S5. Docking score (left) and cytotoxicity (IC_{50}, µM, right) of compounds **1**–**11**. [a]HT-29 human colon cancer cells; [b]MDA-MB-231 human breast cancer cells; [c]HeLa human cervical cancer cells.

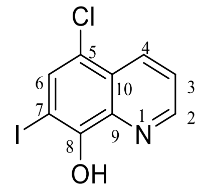

Figure S6. Structure of the FIH-1 inhibitor, clioquinol.

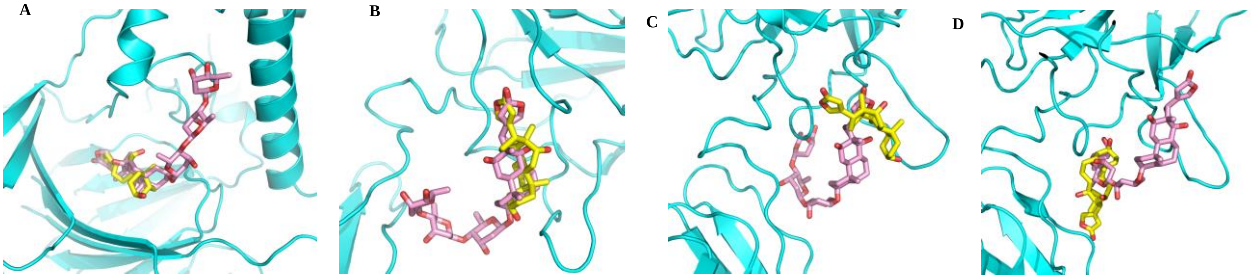

Figure S7. Overlaid docking profiles for digoxin (**2**, pink) and digoxigenin (**10**, yellow) and FIH-1 (A) and the p50 (B), p52 (C), and p65 (D) subunits of NF-κB.

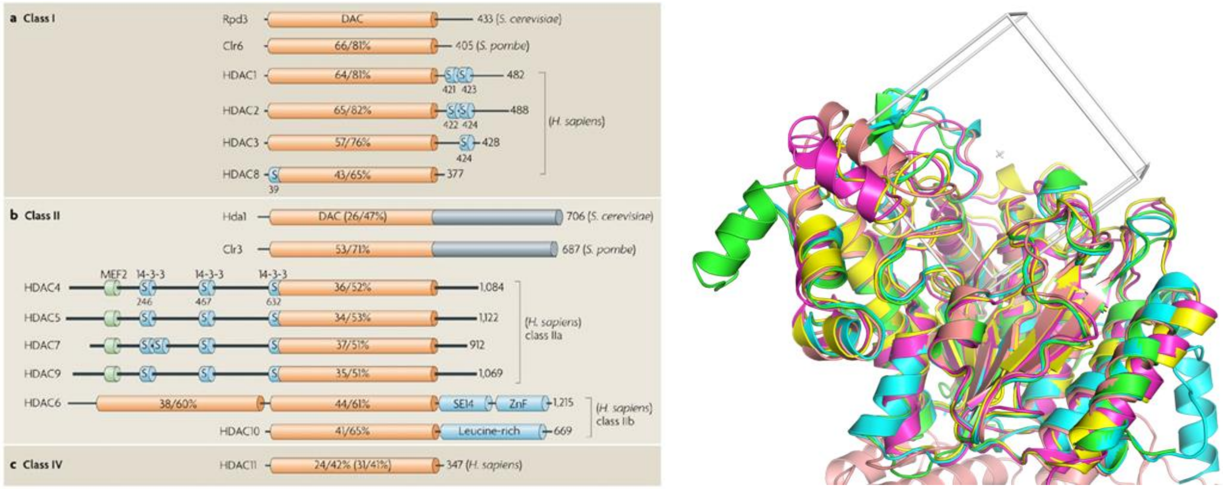

Figure S8. Classes and members (left) and crystal structures (right) of human histone deacetylases (HDACs). The HDAC family has three classes and over 10 members in human. Five crystal structures of different HDACs were used as the receptors for docking.

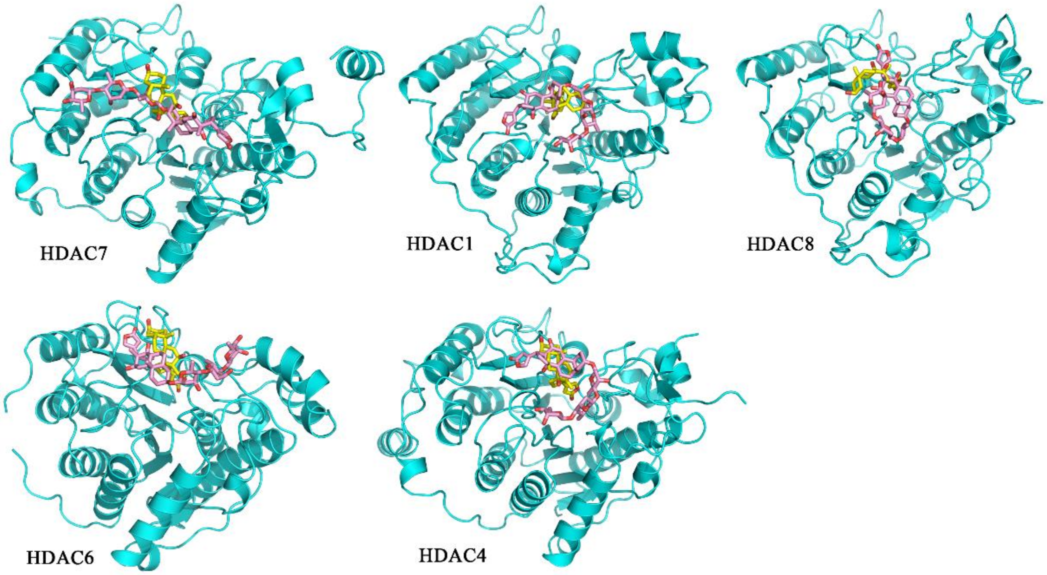

Figure S9. Overlaid docking profiles (overview) for digoxin (**2**, pink) and digoxigenin (**10**, yellow) and HDAC7, HDAC1, HDAC8, HDAC6, and HDAC4.

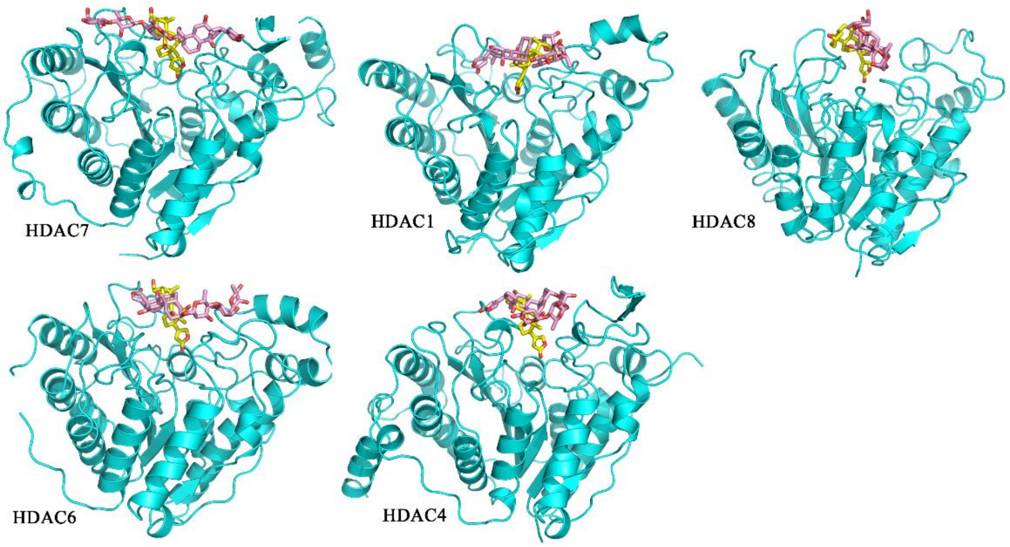

Figure S10. Overlaid docking profiles (sideview) for digoxin (**2**, pink) and digoxigenin (**10**, yellow) and HDAC7, HDAC1, HDAC8, HDAC6, and HDAC4.

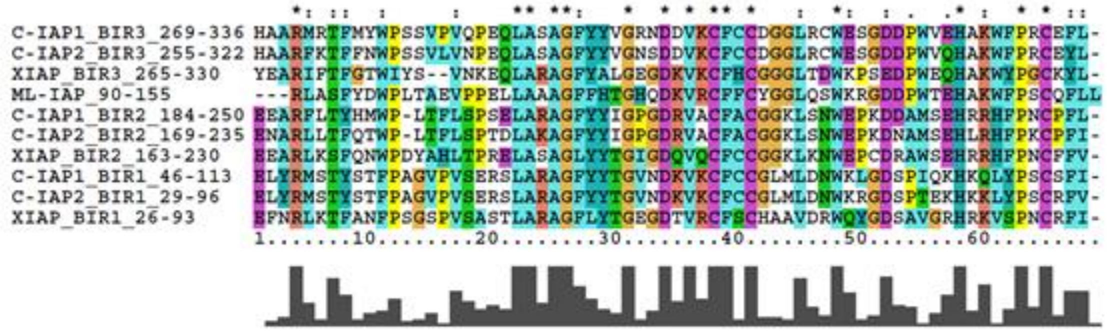

Figure S11. Sequence alignment between cellular inhibitor of apoptosis-1 (cIAP1), cIAP2, X chromosome-linked IAP (XIAP), and melanoma IAP (ML-IAP). The activity domain of IAP proteins is baculoviral IAP repeat (BIR) domain, of which most proteins have more than one BIR domains. The sequence alignment of these BIR domains were generated by Clustal X version 2.0. The active site of the BIR domains have high sequence and structural similarity. Colors correspond to the chemical properties of the residues. Light yellow, aliphatic (P); cyan, polar uncharged (A, F, I, L, M, P, V, and W); yellow, aromatic (P); orange, Glycine (G); red, acidic (K and R); blue, basic (Y); pink, exceptional (C, D, and E); and green, positively charged polar (N, Q, S, and T).

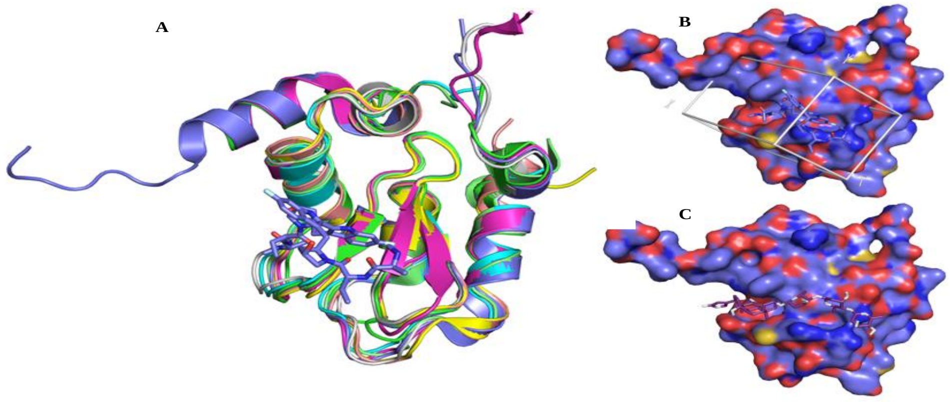

Figure S12. The binding pocket of cIAP1 for small molecules. A. The inhibitor cocrystal structure of cIAP1 (4KMN). B. The binding site of cIAP1 (PDB ID: 4KMN). C. The docking results of digoxin and cIAP1 (4KMN).

The pocket is quite flat and shallow, with two small grooves formed by hydrophobic residues on the bottom and charged residues as the wall. The known cIAP1 inhibitor (Q274543690 could fit fully on the surface of the protein and insert into the grooves.

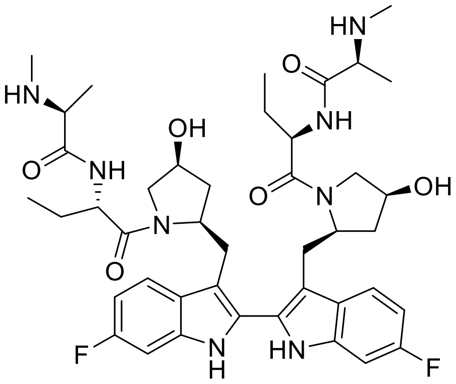

Figure S13. Structure of the cIAP1 inhibitor, Q27454369.

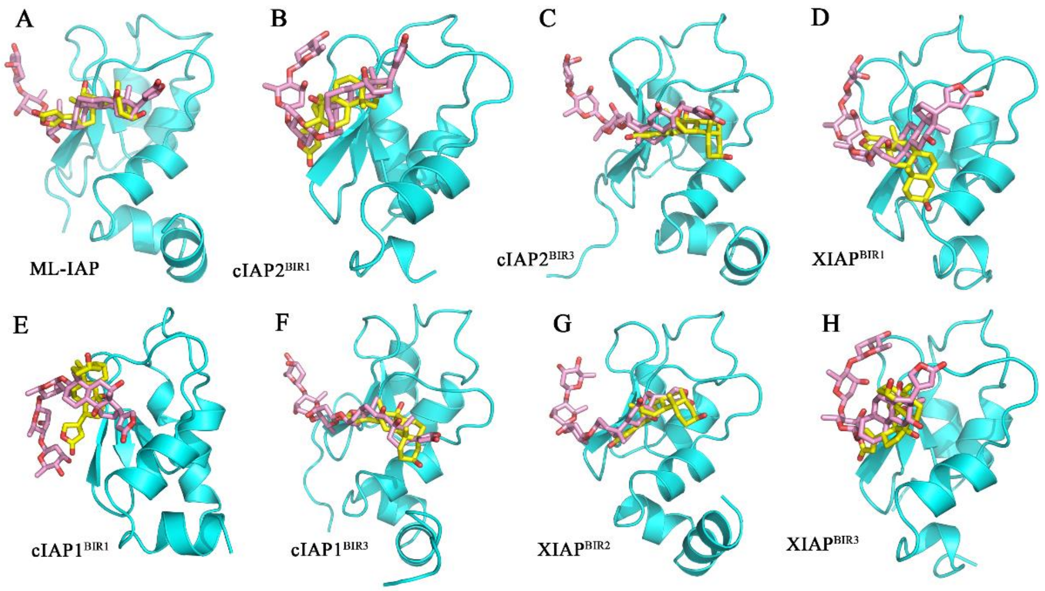

Figure S14. Overlaid docking profiles for digoxin (**2**, pink) and digoxigenin (**10**, yellow) and ML-IAP (A), cIAP2$^{\text{BIR1}}$ (B), cIAP2$^{\text{BIR3}}$ (C), XIAP$^{\text{BIR1}}$ (D), cIAP1$^{\text{BIR1}}$ (E), cIAP1$^{\text{BIR3}}$ (F), XIAP$^{\text{BIR2}}$ (G), XIAP$^{\text{BIR3}}$ (H).

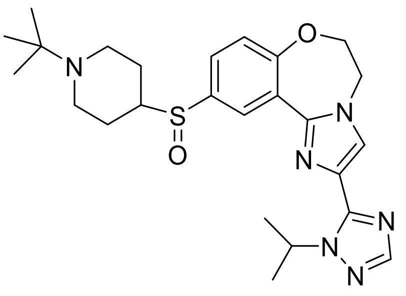

Figure S15. Structure of the PI3K inhibitor,

BWY.

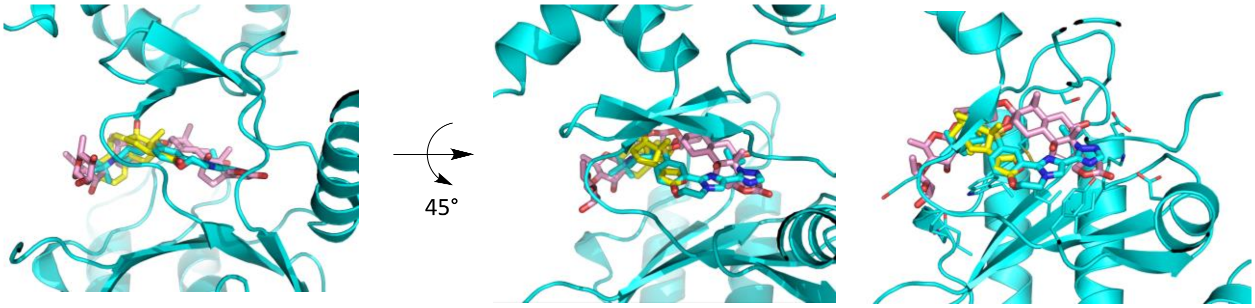

Figure S16. Overlaid docking profiles for digoxin (**2**, pink), digoxigenin (**10**, yellow), and BWY (cyan) and PI3K.
